# Mix Design Optimization of Coal Gangue-Based Geopolymer Foamed Concrete Using Response Surface Methodology

**DOI:** 10.3390/ma18163801

**Published:** 2025-08-13

**Authors:** Dan Wang, Wendong Shan, Rongjie Li, Zhiqiang Song, Lanhui Guo

**Affiliations:** 1 School of Architectural Engineering, Hulunbuir University, Hulunbuir 021008, China; wangdanb@hlbec.edu.cn (D.W.); lirongjie@hlbec.edu.cn (R.L.); songzhiqiang@hlbec.edu.cn (Z.S.); 2 School of Civil Engineering, Inner Mongolia University of Science and Technology, Baotou 014010, China; 3 Engineering Research Center of Safe Mining and Comprehensive Utilization of Mineral Resources of Inner Mongolia Autonomous Region Universities, Hulunbuir 021008, China; 4 School of Civil Engineering, Harbin Institute of Technology, Harbin 150001, China; guolanhui@hit.edu.cn

**Keywords:** foam concrete, geopolymer, response surface methodology, foam content, mix proportion optimization

## Abstract

This study develops a novel geopolymer foamed concrete using coal gangue and slag as precursors, along with a composite alkali activator comprising sodium silicate and sodium hydroxide, based on the physical foaming method. The Box–Behnken Design within Response Surface Methodology was applied to optimize the mix proportions of coal gangue–slag-based geopolymer foamed concrete. The effects of alkali activator dosage, sodium silicate modulus, water-to-binder ratio, and foam content on 28-day compressive strength and thermal conductivity were systematically investigated to determine the optimal mix for achieving a balance between mechanical and thermal performance. Scanning Electron Microscopy and other characterization techniques were used to analyze the microstructural features. The results show that foam content has the most significant influence on both mechanical and thermal performance, while the interaction between sodium silicate modulus and foam content exhibits the most pronounced combined effect. The optimized mix design consists of 9.1% alkali activator dosage, a sodium silicate modulus of 1.07, a water-to-binder ratio of 0.44, and foam content of 50%, resulting in a 28-day compressive strength of 2.30 MPa and thermal conductivity of 0.0781 W/(m·K). The observed performance enhancement is primarily attributed to the increased heterogeneity in the pore structure. This study provides theoretical and technical support for the development of integrated thermal insulation and load-bearing wall materials suitable for severely cold regions.

## 1. Introduction

Coal gangue, a typical aluminosilicate solid waste generated during coal mining and beneficiation, has an annual discharge exceeding 700 million tons. Conventional open-air stockpiling not only occupies vast land resources but also leads to long-term leaching and weathering, which causes the migration and diffusion of heavy metal ions, resulting in persistent contamination of surrounding soils and groundwater systems [1,2]. Foamed concrete, as a mainstream thermal insulation material in the field of building energy efficiency, offers advantages such as lightweight and thermal insulation, but suffers from several critical limitations. These include high energy consumption in cement production, low strength, poor durability, and the incompatibility between mechanical and thermal performance [3,4]. Under the Chinese dual-carbon strategy, *the 14th Five-Year Plan for Building Energy Efficiency and Green Building Development* strongly promotes the integration of thermal insulation and load-bearing technologies, with a mandatory requirement for the large-scale deployment of ultra-low-energy buildings by 2025. This demand is particularly acute in severely cold regions such as Inner Mongolia, where winter heating accounts for up to 20% of total energy consumption [5]. These challenges underscore the urgent need for the development of new-generation solid-waste-based foamed concrete. On one hand, the large-scale utilization of coal gangue is essential for realizing a waste-to-resource circular economy; on the other, it is imperative to overcome performance bottlenecks by developing low-carbon building materials that offer synergistically optimized insulation and load-bearing capabilities under extreme climatic conditions.

Geopolymer materials show great potential in the field of building energy conservation due to their excellent thermal insulation performance and low-carbon characteristics [6,7,8,9]. As one of its typical application forms, the foaming methods used to create foamed concrete mainly include physical foaming, chemical foaming, and composite foaming. Physical foaming has the characteristics of adjustable performance and controllable pore size. Chemical foaming creates a reaction that is difficult to control and can cause an uneven pore size [10]; although the composite foaming can combine the characteristics of the two, the process is complex. In view of the reaction characteristics of geopolymer materials, this study adopts the physical foaming method to ensure the uniformity and stability of the pore structure. Under this technical path, geopolymer foam concrete uses industrial solid wastes such as coal gangue, slag, and fly ash as its primary raw materials, not only significantly reduces the high-carbon emissions associated with traditional cement production [11,12], but also exhibits enhanced mechanical performance through optimization of the activation system [13], thus becoming an ideal alternative material for traditional cement-based foam concrete. At present, the existing research mainly focuses on three key areas: in terms of raw material system optimization, scholars have achieved notable progress through the synergistic utilization of multiple solid wastes. Wang et al. [14] developed a ternary composite system comprising alkali residues, slag, and cement, and prepared alkali residue–slag-based foamed concrete (AG-FC) via a composite foaming process. The resulting material demonstrated excellent overall performance, with a 28-day compressive strength exceeding 1.0 MPa and a durability coefficient of 0.80–0.87, while CO_2_ emissions were reduced by 35–40% compared to traditional foamed concrete. Nandipati et al. [15] pioneered the use of ceramic tile dust in combination with fly ash and slag to develop a ternary alkali-activated foamed block with integrated structural and functional performance. The block achieved a compressive strength of 18.6 MPa, thermal conductivity of 0.38 W/(m·K), and enabled an annual building energy reduction of 4%, with operational cost savings of 5.51%. Research has also shown that using a NaOH–sodium silicate composite activator effectively balances reaction kinetics and gel structure, thereby improving the compressive strength [16]. In terms of performance optimization, Song et al. [17] compared the effects of three representative solid wastes on foamed concrete and, for the first time, quantitatively identified the different contributions of each one: slag had the most significant effect on compressive strength, while furnace slag most effectively reduced thermal conductivity—providing a theoretical basis for function-oriented mix design. Zhang et al. [18] also revealed multi-factor coupling effects, finding that, apart from foam content, other factors exhibited a trend of increasing and then decreasing influence on strength and water absorption. Their optimized foamed concrete was both lightweight and strong, with a favorable pore structure. Dang et al. [19] made process-level breakthroughs by demonstrating that composite foaming agents enhance early-stage foam stability. Under conditions of a modulus of 1.0 and 3% alkali equivalent, foamed geopolymer concrete with excellent early stability was produced, providing key parameters for industrial-scale production. In the area of microstructural mechanism analysis, Wang H et al. [20] used advanced pore characterization techniques to clarify how activator modulus and fly ash content affect pore structure. Lowering the activator modulus increased both porosity and connectivity, resulting in non-uniform pore size distribution, while increasing fly ash content led to a nonlinear trend: porosity decreased up to a 70% replacement rate, beyond which pore distribution became highly uneven. These findings offer important insights for pore structure regulation. Concerning mix design methodology, traditional approaches face clear limitations. Most studies still rely on single-factor experiments [21,22] or orthogonal design methods [23]; however, the former cannot quantify multi-factor interactions, while the latter only identifies discrete level combinations and fails to account for nonlinear interactive effects on performance. In contrast, the Response Surface Methodology (RSM) offers a robust solution by establishing accurate mathematical models to analyze regression equations and identify optimal process parameters, making it well suited for multi-variable problems [24,25]. RSM has been widely used for parameter optimization and reliability analysis [26], overcoming the drawbacks of single-variable and orthogonal methods by employing continuous models and accounting for system error [27]. Because RSM thoroughly considers factor interactions, it has shown outstanding performance in optimizing the mix design of multi-component materials [28,29].

At present, there are two key gaps in the research of coal gangue–slag geopolymer foam concrete: one is the lack of multi-objective collaborative optimization. Most studies only focus on a single performance index and lack a quantitative characterization model for the synergistic effect of thermal insulation and load bearing. Second, the micro–macro correlation is weak, and the relationship between pore structure parameters and macroscopic properties has not been established. In response to these problems, this study proposes two innovations: first, a multi-objective optimization model is established based on the response surface method to break through the limitations of traditional single-objective optimization; secondly, the three-dimensional pore network model and other microscopic means are constructed by industrial CT to reveal the relationship between pore structure and performance. Therefore, in this study, Response Surface Methodology was employed to optimize the mix design of multi-component, multi-objective coal gangue–slag-based geopolymer foamed concrete. The effects of water-to-binder ratio, alkali activator dosage, sodium silicate modulus, and foam content on the material’s mechanical performance (28-day compressive strength) and thermal performance (thermal conductivity) were systematically investigated, and the optimal mix proportions for achieving balanced properties were determined. Furthermore, Scanning Electron Microscopy (SEM) and Mercury Intrusion Porosimetry (MIP) were used to analyze the microstructure, revealing the relationships between porosity, pore size distribution, and macroscopic performance. The findings provide a theoretical foundation for the development of integrated thermal insulation and load-bearing wall materials suitable for severely cold regions.

## 2. Experimental Overview

### 2.1. Raw Materials

**Figure 1:**
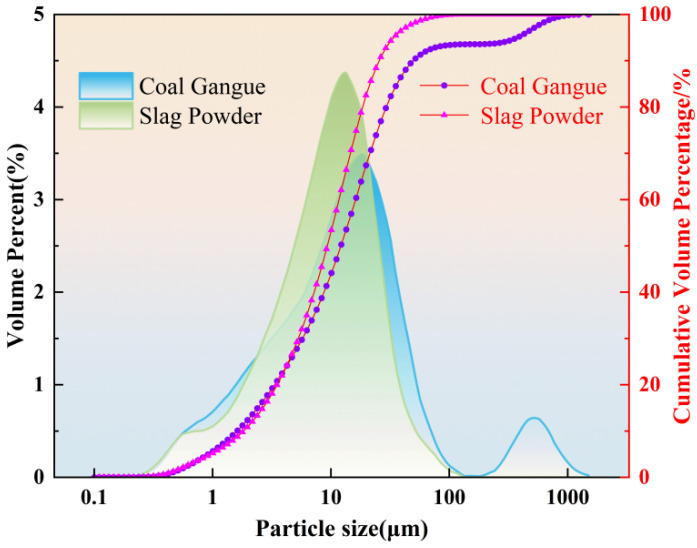
Particle size distribution of coal gangue and slag.

In this study, the coal gangue (CG) used was ground to a fineness of 270 mesh, with a specific surface area of 483 m^2^/kg. The slag powder (SP) was classified as S95-grade, with a specific surface area of 564 m^2^/kg. The chemical composition was determined by XRF (X-ray Fluorescence Spectrometry). The results are shown in Table 1, and their particle size distributions are shown in Figure 1. The foaming agent used was a polymer-based composite cement foaming agent primarily composed of organic components such as α-olefin sulfonate (AOS) and hydroxypropyl methylcellulose (HPMC), with a pH range of 7.5–9.0. The foam stabilizer was a modified silicone-polyether microemulsion produced by Linsen Chemical Co., Ltd. (Linyi, China), with a polyether content of 55% and a pH value ranging from 6.5 to 7.5. The alkali activator was prepared by mixing sodium silicate and NaOH at a specified ratio. The sodium silicate contained 28.3% SiO_2_ and 9.4% Na_2_O, with a modulus of 3.1. The sodium hydroxide (NaOH) used was of analytical grade, with a purity of no less than 96.0%. Tap water was used throughout the experiment.

### 2.2. Experimental Design

A four-factor, three-level Response Surface Optimization was conducted using the Box–Behnken Design (BBD) method using Design-Expert 12 software to evaluate and optimize the properties of coal gangue–slag-based geopolymer foamed concrete. Based on preliminary tests and literature review, the mass ratio of coal gangue to slag was fixed at 1:1 [30]. The foaming method was physical foaming, and the foaming ratio of the foaming agent was set at 1:20. The modulus of the sodium silicate water solution itself was adjusted to the modulus used in the experiment by adding NaOH, that is, the sodium silicate modulus (the molar ratio of SiO_2_ to Na_2_O). Four key variables were selected for analysis: alkali activator dosage (X_1_), sodium silicate modulus (X_2_), water-to-binder ratio (X_3_), and foam content (X_4_). The corresponding response variables were 28-day compressive strength (Y_1_) and thermal conductivity (Y_2_). A total of 29 experimental runs were designed, including 5 center points for model validation. The factor levels for each variable are listed in Table 2.

### 2.3. Experimental Methods

#### 2.3.1. Preparation Procedure and Curing Regime

**Figure 2:**
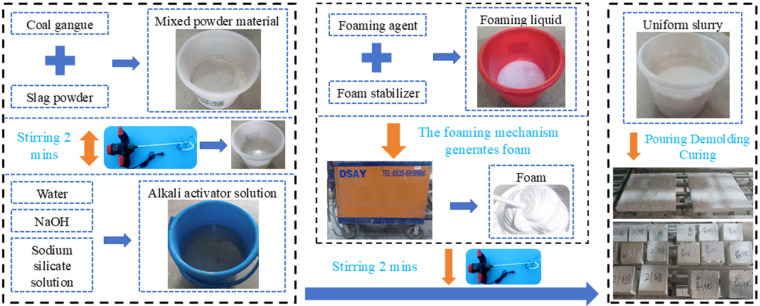
Flowchart of the preparation process for coal gangue–slag geopolymer foam concrete samples.

The coal gangue–slag-based geopolymer foamed concrete was prepared using the physical foaming method. The preparation process, as illustrated in Figure 2, consists of the following major steps: raw material pretreatment, alkali activator solution preparation, foam generation, slurry preparation and foaming, casting, and curing. The detailed procedures are as follows: ① Pretreatment of powdered raw materials: Coal gangue powder and slag powder were weighed according to the designed mass ratio and pre-mixed in a mixing vessel for approximately 2 min to ensure uniform dispersion. This step is critical to avoid uneven component distribution, which could lead to inadequate local activation or fluctuations in material performance. The pretreated powder blend was then used for subsequent activation reactions. ② Preparation of the alkali activator solution: Analytical-grade NaOH was dissolved in water and stirred thoroughly to form a homogeneous alkali activator solution. It was allowed to stand for 24 h until the solution cooled to room temperature before use. ③ Foam generation: The polymer-based composite cement foaming agent (primarily composed of organic components such as α-olefin sulfonate (AOS) and hydroxypropyl methylcellulose (HPMC)) was diluted with water according to the specified ratio and combined with a foam stabilizer to form a stable foam solution. Using a foam generator (Yantai Desai Machinery Manufacturing Co., Ltd. in Shandong, China), dense, uniform, and size-stable foam was produced. ④ Slurry preparation and foam incorporation: The pre-mixed powder from Step ① was combined with the alkali activator solution at the designed water-to-binder ratio and stirred to form a uniform geopolymer slurry. The prepared foam was then added to the slurry and mixed at low speed for 2 min to ensure even foam distribution while minimizing foam collapse. ⑤ Casting, demolding, and curing: The fresh geopolymer foamed slurry was immediately poured into molds, leveled with a trowel, and covered with plastic film to prevent moisture loss and foam degradation. After initial setting, the specimens were demolded and placed in a standard curing environment (temperature 20 ± 2 °C, relative humidity ≥ 95%) for 28 days.

#### 2.3.2. Testing Methods

① Mechanical properties test: Using YAW-300B electro-hydraulic servo hydraulic universal testing machine (Beijing Zhongke Dezhong Technology Co., Ltd. in Beijing, China, according to JG/T 266-2011 ‘Foamed concrete’ standard (foamed concrete product standards focusing on material performance requirements and engineering applications) [31], the compressive strength of coal gangue–slag geopolymer foam concrete cube test block with a size of 100 mm × 100 mm × 100 mm was tested for 28-day compressive strength. The specimens were removed from the standard curing box and placed in the center of the test machine. The specimens were loaded to failure at a rate of 0.8 kN/s, and the failure load was recorded. The above steps were repeated to complete the test for 3 specimens, and the average value was calculated as the final compressive strength.

② Thermal properties test: The thermal conductivity test refers to GB/T 10294-2008 ‘Thermal insulation—Determination of steady-state thermal resistance and related properties—Guarded hot plate apparatus.’ (test method standard for accurate determination of thermal properties of thermal insulation materials) [32]. Specimens measuring 300 mm × 300 mm × 30 mm were used for the steady-state thermal resistance determination.

③ Scanning Electron Microscopy (SEM): A Regulus 8100 scanning electron microscope, manufactured by Hitachi (Tokyo, Japan), was used to observe and analyze the microstructure of the samples. Before the test, the sample was dried and gilded on the surface and adhered to the conductive adhesive for testing. The acceleration voltage was 5–15 kV, the working distance was 5–8 mm, and the high-vacuum mode was used.

④ Mercury Intrusion Porosimetry (MIP): A high-performance, fully automated porosimeter (Model: AutoPore V 9620) produced by Micromeritics (Norcross, GA, USA) was employed to determine the pore structure characteristics of the specimens.

⑤ Industrial Computed Tomography (CT): High-resolution scanning was performed using the X-Mech2000 industrial CT system (225 kV/3 μm), developed by Zhonglian Zhijian, Hangzhou, China. Projection data were collected, and customized image reconstruction algorithms were used to remove artifacts. Three-dimensional rendering and quantitative pore structure analysis were conducted using AVIZO 2023 software.

## 3. Results and Discussion

Based on the 29 experimental runs determined by the BBD, tests were conducted to evaluate the 28-day compressive strength and thermal conductivity of each mixture. The experimental results for both response variables are presented in Table 3. Among them, the 28-day compressive strength varies within the range of 0.7–3.5 MPa, and the thermal conductivity varies within the range of 0.0781–0.1239 W/(m·K). The calculation shows that their average values are 2.07 MPa and 0.1022 W/(m·K), respectively, and the standard deviations are 0.78 MPa and 0.0143 W/(m·K), respectively.

### 3.1. Model Fitting and Accuracy Analysis

Based on the experimental data presented in Table 3, the regression models of Y_1_ and Y_2_ were established by Design-Expert 12 software for multivariate nonlinear regression analysis to evaluate the goodness of fit and significance of the models. The quadratic polynomials are shown in Equations (1) and (2). The goodness-of-fit metrics and Analysis of Variance (ANOVA) results for the models are summarized in Table 4 and Table 5, respectively.

(1) $$\begin{array}{cc} Y_{1}=2.46+0.1750X_{1} & -0.4083X_{2}+0.2750X_{3}-1.03X_{4}-0.1250X_{1}X_{2}+0.0750X_{1}X_{3}+0.0250X_{1}X_{4}\\ & -0.0750X_{2}X_{3}+0.1250X_{2}X_{4}-0.0750X_{3}X_{4}-0.2758X_{1}^{2}-0.2258X_{2}^{2}-0.2008X_{3}^{2}\\ & -0.2508X_{4}^{2} \end{array}$$

(2) $$\begin{array}{cc} Y_{2}=0.1158+0.0048X_{1} & -0.0116X_{2}+0.0047X_{3}-0.0125X_{4}+0.0033X_{1}X_{2}+0.0007X_{1}X_{3}-0.0024X_{1}X_{4}\\ & +0.0038X_{2}X_{3}+0.0060X_{2}X_{4}-0.0017X_{3}X_{4}-0.0066X_{1}^{2}-0.0066X_{2}^{2}-0.0059X_{3}^{2}\\ & -0.0139X_{4}^{2} \end{array}$$

The coefficient of multiple correlation (R^2^) quantifies the strength of the relationship between observed and predicted values, with a value closer to 1 indicating a stronger correlation. The adjusted coefficient of determination (Adjusted R^2^) and the predicted coefficient of determination (Predicted R^2^) assess the model’s fitting accuracy. A difference of less than 0.2 between the Adjusted R^2^ and Predicted R^2^ suggests that the model has high explanatory power. Adequate precision, calculated as the ratio of the difference between the maximum and minimum predicted responses to the average standard deviation of all predicted responses, reflects the model’s robustness against noise. An adequate precision value greater than four indicates that the model is reasonable. Furthermore, if the coefficient of variation (C.V.) is less than 10%, it confirms the reliability of the experimental results [34]. As shown in Table 4, the fitting data of the 28-day compressive strength and thermal conductivity model of coal gangue–slag geopolymer foam concrete all meet the required conditions, indicating that the model has high reliability and fitting accuracy, and can predict 99.7% of the variation in 28-day compressive strength and 99.4% of the variation in thermal conductivity.

According to the Analysis of Variance (ANOVA) results in Table 5, the *p*-values for both response models (Y_1_ and Y_2_) are less than 0.0001, indicating that the models are highly significant. In addition, the lack-of-fit tests were not significant, suggesting that the models exhibit strong predictive accuracy and good overall fit. Table 5 also shows that all four factors have an extremely significant influence (*p* < 0.0001) on both the 28-day compressive strength and thermal conductivity of coal gangue–slag-based geopolymer foamed concrete. Furthermore, significant two-way interaction effects exist between several pairs of variables. Based on the F-values, the interaction between X_2_ and X_4_, as well as between X_1_ and X_2_, has the most pronounced impact on the 28-day compressive strength. For thermal conductivity, the most significant interaction effects are observed between X_2_ and X_4_, and between X_2_ and X_3_.

**Figure 3:**
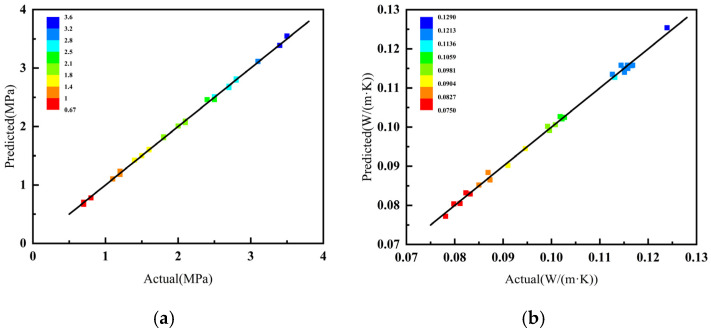
Comparison chart of model predicted values and actual response values. (**a**) Twenty-Eight-day compressive strength; (**b**) thermal conductivity.

The (x, y) scatter diagram is drawn with the model prediction results and the two response value test results as the horizontal and vertical coordinates, and the predicted value and the actual value are compared, as shown in Figure 3. Figures such as 0/4 or 0.07/0.13 in the chart represent the actual and predicted ranges of each performance, respectively. The data points are closely clustered around the reference line (y = x), indicating a high degree of agreement between the predicted and observed results. This demonstrates the strong fitting performance of the models [35]. The minimal deviation observed between experimental and predicted values further validates the accuracy of the ANOVA results, confirming that the models can be reliably used for mixture optimization and performance prediction of coal gangue–slag-based geopolymer foamed concrete.

### 3.2. Effects of Individual Factors and Their Interactions on Response Variables

#### 3.2.1. The 28-Day Compressive Strength

**Figure 4:**
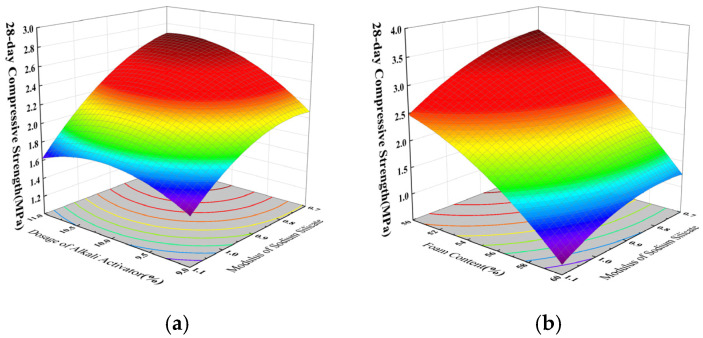
Twenty-eight-day compressive strength response surface curve: (**a**) interaction between X_1_ and X_2_, (**b**) interaction between X_2_ and X_4_.

Based on the results of the regression model ANOVA, and using the Design Expert software to draw the three-dimensional response surface plots, contour plots were generated to analyze the effects of interaction between X_2_ and X_4_, and between X_1_ and X_2_, on the 28-day compressive strength of coal gangue–slag-based geopolymer foamed concrete. The results are illustrated in Figure 4. The colors in the figure represent the magnitude of the 28-day compressive strength. Warmer tones (such as red/orange) indicate higher values, while cooler tones (such as blue/green) indicate lower values.

As shown in Figure 4a, the sodium silicate modulus (X_2_) has a more significant effect on the 28-day compressive strength than the alkali activator dosage (X_1_). A higher modulus reduces the pH value, thereby inhibiting the geopolymerization reaction and decreasing the formation of reaction products, ultimately leading to strength loss. When the modulus is held constant, the compressive strength first increases and then decreases as the alkali activator dosage increases. This is because a moderate dosage promotes the formation of the binding gel phase, while excessive amounts accelerate the reaction too rapidly, hindering subsequent hydration and reducing strength. Figure 4b shows that when the sodium silicate modulus increases from 0.7 to 1.1, the 28-day compressive strength decreases by 28.6%, primarily due to the increased slurry viscosity caused by the higher modulus, which reduces the material’s compactness and degrades the uniformity of the microstructure, leading to reduced strength. Additionally, when the foam content increases from 50% to 60%, the compressive strength drops significantly by 65.7%, which is attributed to the reduction in binder content and the increase in pore volume, which weakens the matrix and thus lowers the compressive strength [36].

#### 3.2.2. Thermal Conductivity

**Figure 5:**
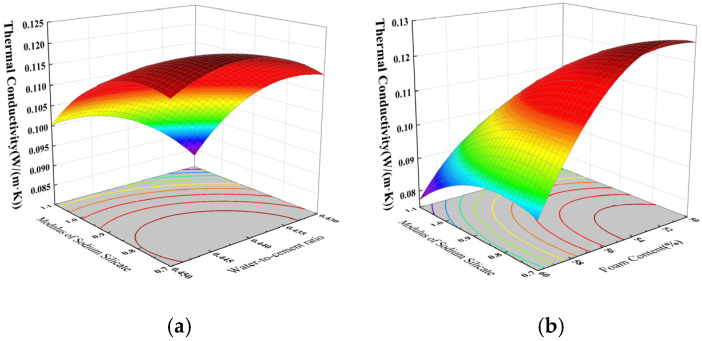
Thermal conductivity response surface curve: (**a**) interaction between X_2_ and X_3_, (**b**) interaction between X_2_ and X_4_.

Based on the results of the regression model ANOVA, and using the Design Expert software to draw the three-dimensional response surface plots, contour plots were generated to analyze the interactive effects of X_2_ and X_4_, as well as X_2_ and X_3_, on the thermal conductivity of coal gangue–slag-based geopolymer foamed concrete. The corresponding results are shown in Figure 5. The colors in the figure indicate the magnitude of the thermal conductivity. Warmer tones (such as red/orange) represent higher values, while cooler tones (such as blue/green) represent lower values.

As shown in Figure 5a, the sodium silicate modulus (X_2_) has a more pronounced effect on thermal conductivity than the water-to-binder ratio (X_3_). The thermal conductivity decreases as the sodium silicate modulus increases, dropping by 28.5% as the modulus increases from 0.7 to 1.1. This is because a low-modulus activator creates a higher-pH environment that destabilizes foam, reducing the number of air voids and, consequently, higher thermal conductivity. When the water-to-binder ratio increases from 0.43 to 0.45, the thermal conductivity decreases by 17%, which can be attributed to improved slurry flowability and better pore structure, resulting in reduced heat transfer. Figure 5b shows that when foam content is held constant, increasing the sodium silicate modulus results alongside a 26.6% reduction in thermal conductivity. Conversely, at a fixed modulus, increasing the foam content leads to a 29.9% decrease in thermal conductivity. This is due to the higher porosity and reduced solid-phase content—since the thermal conductivity of air (0.0259 W/(m·K)) is much lower than that of cement paste (0.5200 W/(m·K)) [37], the increase in air volume interrupts the heat conduction paths, thereby lowering the overall thermal conductivity.

### 3.3. Optimization and Validation of Response Surface Results

**Figure 6:**
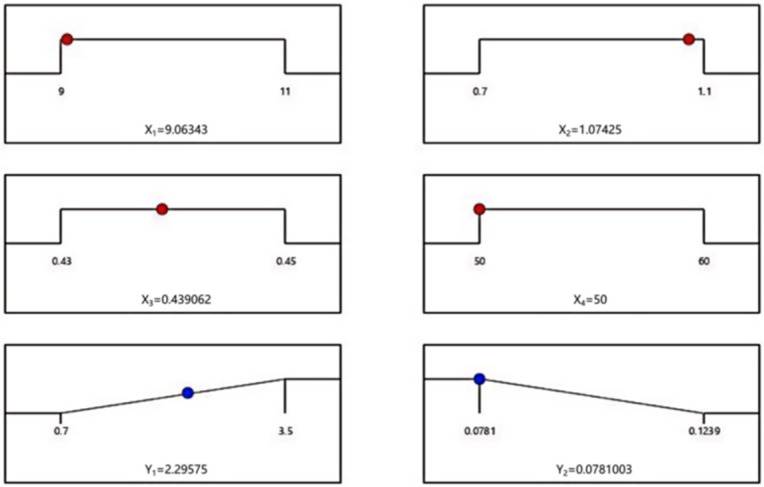
Response surface optimization results.

To achieve a balance between mechanical and thermal performance and obtain optimal overall material properties, the multi-objective optimization function in Design-Expert software was used. The optimization aimed to maximize compressive strength and minimize thermal conductivity based on the developed regression models. The optimal mix design and corresponding optimization results are shown in Figure 6. In the figure, the red circle is the variable, and the blue circle is the response index. The optimal mix proportions determined were as follows: alkali activator dosage of 9.1%, sodium silicate modulus of 1.07, water-to-binder ratio of 0.44, and foam content of 50%. To validate the model’s reliability, three batches of repeated verification experiments were conducted using the optimal formulation. The experimental results are presented in Table 6. The measured values for both compressive strength and thermal conductivity deviated by less than 5% from the predicted values, confirming that the model effectively optimizes the mix proportions of coal gangue–slag-based geopolymer foamed concrete.

### 3.4. Microstructural Analysis

As indicated by Table 3 and Table 5, foam content has the most significant influence on both the thermal conductivity and 28-day compressive strength of coal gangue–slag-based geopolymer foamed concrete. In Group 21 (foam content: 50%), the highest compressive strength was achieved, but the thermal conductivity was also the highest. In contrast, Group 24 (foam content: 60%) exhibited the lowest strength but the lowest thermal conductivity. Among the center point groups, Group 27 (foam content: 55%) achieved both the highest strength and the lowest thermal conductivity. Therefore, foam content was selected as the key variable in this study for in-depth analysis. SEM and related techniques were used to examine pore structure characteristics, including pore size distribution, porosity, and fractal dimension. The objective was to investigate the correlation between microstructural features (porosity, pore morphology, and structural heterogeneity) and macroscopic performance.

**Figure 7:**
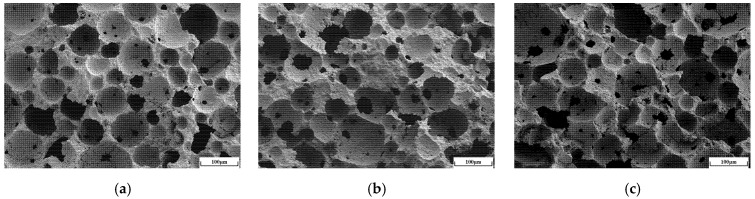
SEM images of geopolymers-based foamed concrete at various foam contents: (**a**) 50% foam content; (**b**) 55% foam content; (**c**) 60% foam content.

**Figure 8:**
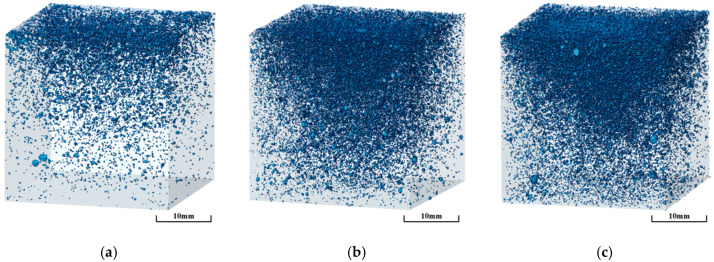
Three-dimensional pore structure renderings of geopolymer foam concrete with different foam dosages: (**a**) 50% foam content; (**b**) 55% foam content; (**c**) 60% foam content.

As clearly shown in Figure 7, when the foam content increases from 50% to 60%, the number of visible pores in the SEM images increases significantly. In addition, pore-related defects such as microcracks and damaged pore walls become more pronounced, while the volume fraction of gel products gradually decreases. These changes directly lead to the formation of more interconnected and through-pores, resulting in a looser, porous material structure. The reduction in gel products weakens the continuity of the matrix, thereby decreasing the compressive strength of the material; meanwhile, the increase in visible pores and the enhancement of interconnected pores significantly reduce the thermal conductivity, as the low thermal conductivity of air dominates the heat transfer path. Three-dimensional pore structure reconstructions of geopolymer foam concrete with varying foam contents were obtained through high-resolution industrial CT scanning, as clearly shown in Figure 8, further revealing the evolution patterns of the pore structure: At a foam content of 50%, the pores were uniformly distributed with smaller sizes. When the foam content increased to 55%, the pore size distribution broadened, showing a clear coexistence of small and large pores. At 60% foam content, a significant number of irregular, large pores formed, and the interconnected pores became notably more pronounced. This structural evolution was consistent with the SEM observations, where a reduction in gel products and an increase in pore wall defects were observed.

**Figure 9:**
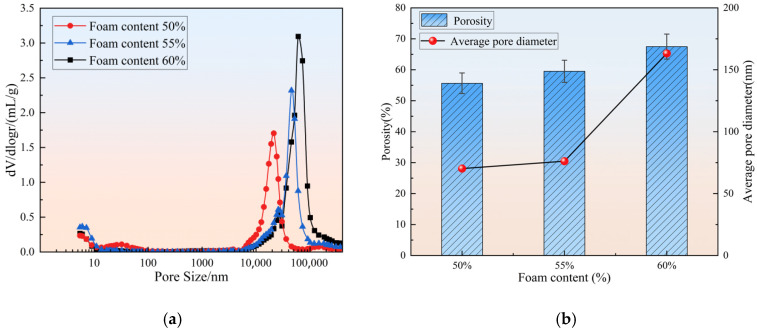
The pore structure of geopolymer foam concrete with different foam content. (**a**) Pore size distribution; (**b**) porosity and average pore diameter.

The MIP results, as shown in Figure 9, indicate that as the foam content increases, the porosity of the specimens significantly increased from 55.64% to 67.49%, while the average pore diameter exhibited a sharp increase from 70.25 nm to 163.26 nm. Notably, at foam contents of 50% and 55%, the porosities were 55.64% and 59.53%, respectively, representing a modest increase of only 6.99%. In contrast, when the foam content increased from 55% to 60%, the porosity jumped from 59.53% to 67.49%, registering a more pronounced increase of 13.37%. A similar trend was observed for the average pore diameter. This phenomenon can be primarily attributed to the distinct bubble stabilization mechanisms at different foam content ranges. Within the 50–55% foam content range, the gel products effectively encapsulate air bubbles, suppressing their coalescence and thereby maintaining relatively stable porosity. However, when the foam content further increases to 60%, the constraining effect of gel products on bubbles weakens significantly, leading to intensified bubble coalescence and enhanced pore connectivity. To quantitatively evaluate the complexity and heterogeneity of the pore network, the box-counting method was employed to calculate the fractal dimension. This method analyzes the power–law relationship between the number of boxes (N(ε)) required to cover the pore space and the box size (ε). The fractal dimension D is defined as follows:

(3) $$D=\underset{\varepsilon \to 0}{\lim}\frac{\log N\left(\varepsilon \right)}{\log\left(\frac{1}{\varepsilon }\right)}$$

A higher fractal dimension indicates a more heterogeneous pore distribution [38]. By dividing the pore network with cubic boxes of various side lengths (ε) and fitting the log-log relationship, the calculated volume-based fractal dimensions for the three specimens with different foam contents were determined to be 1.92 (50%), 2.09 (55%), and 2.16 (60%), respectively. This increasing trend (D_50%_ < D_55%_ < D_60%_) demonstrates that the heterogeneity of the pore structure intensifies with increasing foam content. The fractal dimension exhibited a similar upward trend alongside porosity, suggesting that under high-foam-content conditions, bubble coalescence not only enlarged pore size but, more importantly, intensified the disorder of pore spatial distribution. This behavior can be attributed to the reduction in gel-phase content, which weakens the matrix’s ability to constrain bubble movement. As a result, smaller bubbles more readily coalesce into larger ones, a phenomenon directly reflected by a rightward shift in the pore size distribution curve. Ultimately, this leads to the formation of a larger and more complex pore network.

From the perspective of macroscopic performance, compressive strength shows a clear negative correlation with both porosity and fractal dimension. The experimental data demonstrate that when the porosity increases from 55.6% to 67.5%, the compressive strength decreases by approximately 80%, which is consistent with the Gibson–Ashby theory for cellular materials [39]. This result provides a clear theoretical explanation for the strength reduction mechanism associated with increased porosity. A lower foam content contributes to the formation of a denser and more uniform microstructure, thereby resulting in higher mechanical strength. On the other hand, thermal conductivity decreases as foam content increases. This is primarily due to the higher air-void content, which effectively disrupts heat transfer pathways [40]. This observation is consistent with the findings of Jaya N. A. et al. [41]. These results demonstrate that precise control of foam content can effectively tailor the pore structure characteristics, enabling the synergistic optimization of both mechanical and thermal performance in coal gangue–slag-based geopolymer foamed concrete.

Compared with previous studies, the optimized coal gangue–slag-based geopolymer foam concrete in this work demonstrates remarkable advantages in both thermal conductivity (0.0781 W/(m·K)) and compressive strength (2.30 MPa). For instance, Wang et al. [6] reported that alkali residue-slag-based foam concrete achieved only 1.35–2.02 MPa compressive strength, while conventional cement-based foam concrete typically exhibits 0.15–0.45 W/(m·K) thermal conductivity and 0.5–1.2 MPa compressive strength [42]. The developed material significantly enhances mechanical performance while maintaining low thermal conductivity, providing a superior solution for building wall materials in severely cold regions.

## 4. Conclusions

In this study, RSM was employed to optimize the mix design of coal gangue–slag-based geopolymer foamed concrete, to investigate the synergistic effects of raw material proportions on both mechanical and thermal performance. Through an integrated approach combining microstructural characterization techniques—including SEM and industrial CT—the underlying mechanisms governing material performance were elucidated from a microstructural perspective. The main conclusions are as follows:

1. The optimal mix proportion was determined to be 9.1% alkali activator dosage, sodium silicate modulus of 1.07, water-to-binder ratio of 0.44, and 50% foam content. Under these conditions, the 28-day compressive strength reached 2.30 MPa while maintaining a low thermal conductivity of 0.0781 W/(m·K). Compared with other geopolymer foam concretes and conventional cement-based foam concretes, the developed material demonstrates significantly enhanced thermal insulation performance without compromising mechanical strength. Furthermore, the utilization of solid waste materials effectively reduces carbon emissions, meeting the technical requirements for building wall materials in severely cold regions.
2. A second-order regression model was developed based on the BBD to relate four key factors—alkali activator dosage, sodium silicate modulus, water-to-binder ratio, and foam content—to two performance indicators: 28-day compressive strength and thermal conductivity. The model exhibited high reliability, with R^2^ values exceeding 0.99 and prediction errors below 5%, demonstrating its applicability for optimizing the mix design of coal gangue–slag-based geopolymer foamed concrete.
3. Among the single-factor effects, foam content had the most significant impact on both 28-day compressive strength and thermal conductivity. Regarding two-factor interactions, the interaction between sodium silicate modulus and foam content exerted the most pronounced influence on both performance indicators, with a significantly greater effect than other interaction combinations.
4. As the foam content increases, the reduction in gel product content promotes the coalescence of small bubbles, resulting in a complex and disordered pore structure. This structural disorder leads to a decline in mechanical strength, while the increased porosity effectively enhances thermal performance. The study quantitatively reveals the correlation between pore structure parameters and macroscopic properties, providing important theoretical support for the development of thermal insulation building materials.

Although this study has achieved promising results in the mix proportion optimization of coal gangue–slag-based geopolymer foam concrete, several limitations should be acknowledged. Firstly, the tested parameter ranges remain relatively limited; future studies could expand the research scope to explore a wider combination of mix proportions. Secondly, the current work only evaluated 28-day mechanical and thermal properties, lacking long-term durability assessments. Subsequent research should incorporate comprehensive long-term performance evaluations. Furthermore, this study was conducted under laboratory conditions; practical engineering environments should be considered in future work to further validate material applicability. Addressing these aspects will provide more comprehensive theoretical and technical support for industrial-scale applications of this material.

## Figures and Tables

**Table 1:** The chemical composition of coal gangue and slag.

Material	Mass Fraction/%							
	SiO_2_	Al_2_O_3_	CaO	FeO	Fe_2_O_3_	MgO	TiO_2_	Others
Coal Gangue	51.88	44.80	0.15	/	0.42	0.09	2.33	0.33
Slag Powder	38.2	7.76	39.7	1.09	/	11.02	/	2.23

**Table 2:** Factors and levels in the Box–Behnken Design.

Factors	Code	Levels		
		−1	0	+1
Alkali Activator Dosage	X_1_	9%	10%	11%
Sodium Silicate Modulus	X_2_	0.7	0.9	1.1
Water-to-binder ratio	X_3_	0.43	0.44	0.45
Foam Content	X_4_	50%	55%	60%

**Table 3:** Results of response surface experiments with various mix proportions.

Test Number	Alkali Activator Dosage/%	Sodium Silicate Modulus	Water-to-Binder Ratio	Foam Content/%	28-Day Compressive Strength/MPa	Thermal Conductivity/W/(m·K)
	X_1_	X_2_	X_3_	X_4_	Y_1_	Y_2_
1	9	0.7	0.44	55	2.1	0.1131
2	11	0.7	0.44	55	2.7	0.1165
3	9	1.1	0.44	55	1.5	0.0832
4	11	1.1	0.44	55	1.6	0.0996
5	10	0.9	0.43	50	2.7	0.1022
6	10	0.9	0.45	50	3.4	0.1150
7	10	0.9	0.43	60	0.8	0.0811
8	10	0.9	0.45	60	1.2	0.0873
9	9	0.9	0.44	50	2.8	0.1008
10	11	0.9	0.44	50	3.1	0.1158
11	9	0.9	0.44	60	0.7	0.0798
12	11	0.9	0.44	60	1.1	0.0850
13	10	0.7	0.43	55	2.1	0.1151
14	10	1.1	0.43	55	1.4	0.0823
15	10	0.7	0.45	55	2.8	0.1166
16	10	1.1	0.45	55	1.8	0.0992
17	9	0.9	0.43	55	1.6	0.0946
18	11	0.9	0.43	55	1.8	0.1018
19	9	0.9	0.45	55	2.0	0.1027
20	11	0.9	0.45	55	2.5	0.1126
21	10	0.7	0.44	50	3.5	0.1239
22	10	1.1	0.44	50	2.5	0.0910
23	10	0.7	0.44	60	1.2	0.0869
24	10	1.1	0.44	60	0.7	0.0781
25	10	0.9	0.44	55	2.5	0.1165
26	10	0.9	0.44	55	2.5	0.1168
27	10	0.9	0.44	55	2.5	0.1144
28	10	0.9	0.44	55	2.4	0.1157
29	10	0.9	0.44	55	2.4	0.1158

**Table 4:** Analysis of response model fitness.

Response	R^2^	Adjusted R^2^	Predicted R^2^	Adequate Precision	C.V./%
Y_1_	0.9987	0.9974	0.9955	100.5503	1.92
Y_2_	0.9969	0.9939	0.9848	59.4500	1.10

**Table 5:** Analysis of Variance (ANOVA) for the regression model.

Response Variable	Source	Sum of Squares	Mean Square	F-Value	*p*-Value	Significance
Y_1_	Model	17.04	1.22	774.71	<0.0001	Significant
	X_1_	0.3675	0.3675	233.86	<0.0001	
	X_2_	2.00	2.00	1273.26	<0.0001	
	X_3_	0.9075	0.9075	577.50	<0.0001	
	X_4_	12.61	12.61	8022.95	<0.0001	
	X_1_X_2_	0.0625	0.0625	39.77	<0.0001	
	X_1_X_3_	0.0225	0.0225	14.32	0.0020	
	X_1_X_4_	0.0025	0.0025	1.59	0.2278	
	X_2_X_3_	0.0225	0.0225	14.32	0.0020	
	X_2_X_4_	0.0625	0.0625	39.77	<0.0001	
	X_3_X_4_	0.0225	0.0225	14.32	0.0020	
	X_1_^2^	0.4935	0.4935	314.06	<0.0001	
	X_2_^2^	0.3308	0.3308	210.52	<0.0001	
	X_3_^2^	0.2616	0.2616	166.49	<0.0001	
	X_4_^2^	0.4081	0.4081	259.71	<0.0001	
	Residual	0.0220	0.0016			
	Lack of fit	0.0100	0.0100	0.3333	0.9277	Not significant
Y_2_	Model	0.0058	0.0004	324.8	<0.0001	Significant
	X_1_	0.0003	0.0003	213.79	<0.0001	
	X_2_	0.0016	0.0016	1261.46	<0.0001	
	X_3_	0.0003	0.0003	207.84	<0.0001	
	X_4_	0.0019	0.0019	1485.23	<0.0001	
	X_1_X_2_	0	0	33.25	<0.0001	
	X_1_X_3_	1.823	1.823	1.43	0.2510	
	X_1_X_4_	0	0	18.89	0.0007	
	X_2_X_3_	0.0001	0.0001	46.65	<0.0001	
	X_2_X_4_	0.0001	0.0001	114.26	<0.0001	
	X_3_X_4_	0	0	8.57	0.0110	
	X_1_^2^	0.0003	0.0003	220.59	<0.0001	
	X_2_^2^	0.0003	0.0003	220.59	<0.0001	
	X_3_^2^	0.0002	0.0002	180.64	<0.0001	
	X_4_^2^	0.0013	0.0013	986.03	<0.0001	
	Residual	0	1.271			
	Lack of fit	0	1.434	1.66	0.3299	Not significant

Note: A *p*-value less than 0.05 indicates statistical significance, while a *p*-value of less than 0.001 indicates high relevance. A larger F-value implies stronger significance [33].

**Table 6:** Prediction and validation of experimental results.

Numerical Values	28-Day Compressive Strength/MPa	Thermal Conductivity/W/(m·K)
Predicted values	2.30	0.0781
Average value	2.23	0.0819
Error/%	3.04	4.82

## Data Availability

The original contributions presented in this study are included in the article. Further inquiries can be directed to the corresponding author.

## References

[1] Qiu J., Zhu M., Zhou Y., Guan X. (2021). Effect and mechanism of coal gangue concrete modification by fly ash. Constr. Build. Mater..

[2] Mischinenko V., Vasilchenko A., Lazorenko G. (2024). Effect of Waste Concrete Powder Content and Microwave Heating Parameters on the Properties of Porous Alkali-Activated Materials from Coal Gangue. Materials.

[3] Wang X., Wu Y., Li X., Li Y., Tang W., Dan J., Hong C., Wang J., Yang X. (2024). Effect of Triterpenoid Saponins as Foaming Agent on Mechanical Properties of Geopolymer Foam Concrete. Materials.

[4] Xin Q., Lu S., Shao P., Zhang H., Zhang M. (2025). The enhancement of compressive strength and copper ion adsorption of foamed concrete by recycled glass fiber reinforced plastic powder. Constr. Build. Mater..

[5] Zhang N., Liu G., Man X., Wang Q. (2025). Climate and resource characteristics-based zoning of heat source retrofit for heating systems and implications for heat consumption of urban settlements in cold regions of China. Energy.

[6] Lo Bianco A., Calvino M., Cavallaro G., Lisuzzo L., Pasbakhsh P., Milioto S., Lazzara G., Lvov Y. (2024). Flame-resistant inorganic films by self-assembly of clay nanotubes and their conversion to geopolymer for CO2 capture. Small.

[7] Ren J., Matar M., Tonin N., Pu C., White C., Srubar W. (2025). Understanding the effects of hematite and brucite additions on fresh-and hardened-state properties of metakaolin-based geopolymer cements. Appl. Clay Sci..

[8] Shen R., Li X., Li S. (2025). Study on Mechanical Properties of Alkali-Activated Coal Gasification Slag Concrete. Materials.

[9] Calvino M.M., Lisuzzo L., Cavallaro G., Lazzara G., Milioto S. (2022). Halloysite-based geopolymers filled with wax microparticles as sustainable building materials with enhanced thermo-mechanical performances. J. Environ. Chem. Eng..

[10] Korniejenko K., Pławecka K., Bazan P., Figiela B., Kozub B., Mróz K., Łach M. (2023). Green building materials for circular economy—Geopolymer foams. Proc. Eng. Technol. Innov..

[11] Yang X., Wu S., Xu S., Chen D., Zhao Z., Chen B., Liang X. (2024). Development of a novel emulsified asphalt enhanced steel slag-based geopolymer foamed concrete. Constr. Build. Mater..

[12] Bazan P., Figiela B., Kozub B., Łach M., Mróz K., Melnychuk M., Korniejenko K. (2024). Geopolymer Foam with Low Thermal Conductivity Based on Industrial Waste. Materials.

[13] Hao Y., Yang G., Liang K. (2022). Development of fly ash and slag-based high-strength alkali-activated foam concrete. Cem. Concr. Compos..

[14] Wang Z., Wu K., Liu S., Li M., Zhang X., Yuan Z. (2025). Low-Carbon Foamed Concrete Based on Alkali Residue and GGBS versus Conventional Foamed Concrete: Comparative Experimental Research. J. Mater. Civ. Eng..

[15] Nandipati S., Degloorkar N., Pullagura G., Barik D., Paramasivam P., Althaqafi E., Islam S., Al-Sareji O. (2025). Evaluating energy consumption patterns in novel foamed ternary alkali-activated masonry blocks. Sci. Rep..

[16] Huang G., Zhang X., Liu M., Fang B., Wang C., Mi H. (2023). Compatibility of sodium hydroxide, sodium silicate and calcium-enriched additives in alkali-activated materials: From the perspectives of flowability, strength and microstructure. Constr. Build. Mater..

[17] Song Q., Bao J., Xue S., Zhang P., Mu S. (2021). Collaborative disposal of multisource solid waste: Influence of an admixture on the properties, pore structure and durability of foam concrete. J. Mater. Res. Technol..

[18] Zhang S., Qi X., Guo S., Zhang L., Ren J. (2022). A systematic research on foamed concrete: The effects of foam content, fly ash, slag, silica fume and water-to-binder ratio. Constr. Build. Mater..

[19] Dang J., Tang X., Xiao J., Duan Z., Han A. (2023). Early Stability Behavior and Mechanism of Alkali-Activated Foamed Concrete. J. Build. Mater..

[20] Wang H., Gao S., Meng Z., Wu Y., Liu X. (2024). Micro-pore Structure and Macro-properties of Fly Ash-Slag Based Foam Geopolymer. J. Build. Mater..

[21] Rong X., Zhang X., Zhang J., Xu W., Zhang Z. (2023). Study on mechanical and thermal properties of alkali-excited fly ash aerogel foam concrete. Constr. Build. Mater..

[22] Pantongsuk T., Kittisayarm P., Muenglue N., Benjawan S., Thavorniti P., Tippayasam C., Nilpairach S., Heness G., Chaysuwan D. (2021). Effect of hydrogen peroxide and bagasse ash additions on thermal conductivity and thermal resistance of geopolymer foams. Mater. Today Commun..

[23] Cao D., Zhang H., Dong H., Zhao Y. (2025). Effect of reactive magnesia on dry shrinkage of geopolymer concrete and its bond property with BFRP bar. Constr. Build. Mater..

[24] Gan Y., Li C., Chen A., Li Y., Wu S. (2022). A model of pyrolysis carbon black and waste chicken feather using a response surface method in hot-mix asphalt mixtures. J. Mater. Civ. Eng..

[25] Zhang L., Zhai J. (2021). Application of response surface methodology to optimize alkali-activated slag mortar with limestone powder and glass powder. Struct. Concr..

[26] Feng Z., Wu X., Chen H., Qin Y., Zhang L., Skibniewski M. (2022). An energy performance contracting parameter optimization method based on the response surface method: A case study of a metro in China. Energy.

[27] Wang X., Zhang Y., Zhao W., Wang Z., Wang Z., Wang Y. (2023). Research on optimizing performance of new slurries for EPBS soil conditioning based on response surface method. Constr. Build. Mater..

[28] Chelladurai S., Murugan K., Ray A., Upadhyaya M., Narasimharaj V., Gnanasekaran S. (2021). Optimization of process parameters using response surface methodology: A review. Mater. Today Proc..

[29] Maaze M., Shrivastava S. (2023). Design optimization of a recycled concrete waste-based brick through alkali activation using Box-Behnken design methodology. J. Build. Eng..

[30] Shi Q., Zhou M., Bai J., Zhang K., Li D. (2025). Influence of coal gangue on the properties of coal-based solid waste geopolymer grouting material. J. Build. Mater..

[31] (2011). Foamed Concrete.

[32] (2008). Thermal Insulation-Determination of Steady-State Thermal Resistance and Related Properties-Guarded Hot Plate Apparatus.

[33] Aziz A., Driouich A., Felaous K., Bellil A. (2023). Box-Behnken design-based optimization and characterization of new eco-friendly building materials based on slag activated by diatomaceous earth. Constr. Build. Mater..

[34] Abdulkadir I., Mohammed B., Liew M., Wahab M. (2021). Modelling and multi-objective optimization of the fresh and mechanical properties of self-compacting high volume fly ash ECC (HVFA-ECC) using response surface methodology (RSM). Case Stud. Constr. Mater..

[35] Zhou Y., Xie L., Kong D., Peng D., Zheng T. (2022). Research on optimizing performance of desulfurization-gypsum-based composite cementitious materials based on response surface method. Constr. Build. Mater..

[36] Ren C., Wang L., Kong D., Yang R., Wang Y., Tian Y., Tao T. (2025). Performance study and effect mechanism of red mud manufactured sand foam concrete using a single-factor experiment. Constr. Build. Mater..

[37] Fu X., Chung D. (1997). Effects of silica fume, latex, methylcellulose, and carbon fibers on the thermal conductivity and specific heat of cement paste. Cem. Concr. Res..

[38] Ding K., Zeng C. (2025). Study on the compressive strength, pore structure characteristics, and fractal dimension of the ecological porous concrete specimens based on ordinary Portland cement. Constr. Build. Mater..

[39] Yu G., Li Z., Li S., Zhang Q., Hua Y., Liu H., Zhao X., Dhaidhai D., Li W., Wang X. (2020). The selection of internal architecture for porous Ti alloy scaffold: A compromise between mechanical properties and permeability. Mater. Des..

[40] Liu C., Liu G. (2021). Characterization of pore structure parameters of foam concrete by 3D reconstruction and image analysis. Constr. Build. Mater..

[41] Jaya N., Liew Y., Heah C., Abdullah M., Hussin K. (2020). Correlation between pore structure, compressive strength, and thermal conductivity of porous metakaolin geopolymer. Constr. Build. Mater..

[42] Zhao D., Xu J., Han Z., Liu Y., Liu Y., Yang X. (2024). Study on the correlation between pore structure characterization and early mechanical properties of foamed concrete based on X-CT. Constr. Build. Mater..

